# Cost analysis and critical success factors of the use of oxygen concentrators versus cylinders in sub-divisional hospitals in Fiji

**DOI:** 10.1186/s12913-021-06687-8

**Published:** 2021-07-02

**Authors:** Susan McAllister, Louise Thorn, Sainimere Boladuadua, Mireia Gil, Rick Audas, Tim Edmonds, Eric Rafai, Philip C. Hill, Stephen R. C. Howie

**Affiliations:** 1 Centre for International Health, Department of Preventive and Social Medicine, Dunedin School of Medicine, University of Otago, PO Box 56, Dunedin, New Zealand; 2 Cure Kids Fiji, Suva, Fiji; 3 Department of Paediatrics: Child & Youth Health, University of Auckland, Auckland, New Zealand; 4 Azimut 360 SCCL, Barcelona, Spain; 5 Faculty of Medicine, Memorial University of Newfoundland, St John’s, Canada; 6 Cure Kids, Auckland, New Zealand; 7 Ministry of Health and Medical Services, Suva, Fiji

**Keywords:** Oxygen concentrators, Cost, Fiji

## Abstract

**Background:**

Oxygen is vital in the treatment of illnesses in children and adults, yet is lacking in many low and middle-income countries health care settings. Oxygen concentrators (OCs) can increase access to oxygen, compared to conventional oxygen cylinders. We investigated the costs and critical success factors of OCs in three hospitals in Fiji, and extrapolated these to estimate the oxygen delivery cost to all Sub-Divisional hospitals (SDH) nationwide.

**Methods:**

Data sources included key personnel interviews, and data from SDH records, Ministry of Health and Medical Services, and a non-governmental organisation. We used Investment Logic Mapping (ILM) to define key issues. An economic case was developed to identify the investment option that optimised value while incorporating critical success factors identified through ILM. A fit-for-purpose analysis was conducted using cost analysis of four short-listed options. Sensitivity analyses were performed by altering variables to show the best or worst case scenario. All costs are presented in Fijian dollars.

**Results:**

Critical success factors identifed included oxygen availability, safety, ease of use, feasibility, and affordability. Compared to the status quo of having only oxygen cylinders, an option of having a minimum number of concentrators with cylinder backup would cost $434,032 (range: $327,940 to $506,920) over 5 years which would be 55% (range: 41 to 64%) of the status quo cost.

**Conclusion:**

Introducing OCs into all SDHs in Fiji would reduce overall costs, while ensuring identified critical success factors are maintained. This study provides evidence for the benefits of OCs in this and similar settings.

**Supplementary Information:**

The online version contains supplementary material available at 10.1186/s12913-021-06687-8.

## Background

Every year, over five million children die globally; a large proportion of these deaths occur in low and middle-income countries (LMICs), and most are preventable [1]. Pneumonia is a leading cause of death in both adults and children [1, 2], and a low level of blood oxygen (hypoxaemia) is a major complication of pneumonia [3]. Detecting and treating severe pneumonia with oxygen reduces death [4, 5]. Hypoxaemia also occurs in severe newborn conditions and other illnesses, and in the four leading causes of adult death globally, namely ischaemic heart disease, stroke, lower respiratory infections and chronic obstructive pulmonary disease [6]. Oxygen therapy is, therefore, an essential component of treatment in preventing morbidity and mortality in children as well as in adults, and is included in the World Health Organization (WHO) list of essential medicines [7]. Despite being essential, reliable access to medical oxygen is often lacking in LMICs, frequently due to cost and logistical difficulties [3].

Oxygen cylinders have been the standard form of oxygen supply in most health facilities worldwide. They are expensive, heavy and difficult to transport, and require regular replenishment, all of which are barriers in LMICs [8]. There is increasing experience in the use of oxygen concentrators to successfully supply oxygen to patients in hospitals and smaller health facilities in LMICs [9–15]. Portable concentrators can provide a reliable source of oxygen at a maximum rate of 5–10 l per minute [14]. With basic technical support they can run for many years [16].

In Fiji, with a population of approximately 889,327 [17] people spread across one-third of the country’s 332 islands, health services are split into four Divisional Health Services and further separated into sub-divisions. Public health services are provided through 19 Sub-Divisional Hospitals (SDH), Health Centres (HC), and Nursing Stations (NS). The Fiji Ministry of Health and Medical Services (MoHMS) allocates a budget to SDHs that includes leasing costs for oxygen cylinders for the hospital, HCs and NSs within its sub-division. The Fiji Islands Oxygen Project (FiO_2_) has the ultimate goal of achieving universal access to oxygen in Fiji. Oxygen concentrators were installed in three pilot sites (Nausori Health Centre, Taveuni Hospital, and Nabouwalu Hospital) between March 2016 and August 2018. The costs and benefits of concentrator installation at SDHs in these sites were investigated to determine whether there is a case for further investment into an oxygen concentrator-based solution for oxygen delivery to SDHs in Fiji, and in similar settings.

## Methods

An initial scoping visit to the three pilot sites was followed up by visits to each site to facilitate the collection of cost data. Data from the three sites were extrapolated to provide a cost estimate for expansion to the 16 remaining SDHs and the ongoing costs for all 19 SDHs over a 5-year period which is the approximate time estimated for concentrators to function before needing replacement.

### Key data and data sources

Multiple sources were used to collect data. The risks and benefits of cylinder versus oxygen concentrator use were documented through discussions with key clinical and administrative staff. The main discussion questions were related to ease of use, safety issues, training, costs, supply, and perceived benefits and difficulties, with discussions following along themes and issues raised by staff (Additional file 1). Cylinder costs and ongoing refill, rental and transport costs were obtained from SDH administrative records. Cylinder equipment costs were sourced from the Fiji Pharmaceutical and Biomedical Services, the Fiji government’s health procurement service. Concentrator capital expenditure (concentrator, spare parts, shipping and solar system) was obtained from Cure Kids New Zealand and the concentrator manufacturer’s website. The ongoing costs for electricity and biomedical engineering support were obtained from SDH administrative records. Information collected was at the institutional level (further details are documented in Additional file 2). No individual participants were recruited or data collected for this study.

### Analysis

An investment logic mapping (ILM) [18] exercise was used to define the central issues expressed by key personnel (Additional file 3). ILM is a method designed to ensure that robust discussion and thinking is done before any proposed investment [18]. An economic case was then developed to identify the investment option that optimised value for money while incorporating the critical success factors identified through the ILM. We then conducted a fit-for-purpose analysis of four short-listed options: 1) cylinders only (status quo); 2) concentrators only; 3) minimum number of concentrators with cylinder back-up; 4) number of concentrators convenient to clinical staff, with cylinder back-up. The minimum number of concentrators was based on the World Health Organization (WHO) guideline of one concentrator per 15 beds and an additional spare concentrator per facility [19]. An appraisal period of 5 years was estimated as this is a reasonable conservative working estimate for the life span of an oxygen concentrator. All costs are presented in Fijian dollars (FJD) which averaged United States (US) dollars 0.463 for the year 2019 [20]. Details of assumptions used are described in Additional file 2.

#### Analysis of monetary costs and critical success factors

All options were compared to Option One (status quo). Present value (PV) of monetary benefits is the current value of a future sum of cash flows, discounted at a ‘discount rate’. For this project, there were no incoming cash flows, so we used the savings compared to the existing operating costs. The formula of *R*_*t*_ / (1 + *i*)^*t*^ was used for calculating the PV of monetary benefits. *R*_*t*_ was the savings in operating costs per year. The operating costs per year for each of the four options were calculated by dividing the operating costs by five. The savings in operating costs were then calculated by subtracting the yearly operating costs for each option from the Option One yearly operating costs. The discount rate per year (*i*) was 4% in New Zealand which was therefore used in the calculation [21]. The number of years post installation (*n*) was 1 for the first year, 2 for the second, and so on. The PV was calculated for each year of the appraisal, which in this case is five. The sum of all five equations equals the total PV.

Present value of costs (PVC) is the capital costs spent at the start of the project. As Option One also has capital costs, the present value of costs is the difference between capital costs for Option One and the remaining three options (two to four), as these are extra costs over and above the existing system. Net present value (NPV) is the benefits minus the costs (NPV = PV (benefits) – PV (Costs)). NPV was calculated by subtracting the present value of monetary benefits from the present value of costs.

#### Sensitivity analyses

Sensitivity analyses were performed by altering variables to show the best or worst case scenarios of the costs for the proposed expansion. The costs were altered from the preferred option for expansion. The estimated cost of the preferred option, and the best and worst-case costs, were compared to the costs for the existing oxygen system to determine if any of the changes to the variables made the preferred option no longer affordable.

## Results

Three key issues were identified from the investment logic mapping exercise:

1. The SDHs and their dependent smaller facilities have oxygen supplies, however, they often need to ration oxygen in case of an emergency, and sometimes completely run out due to refill stations being far from the SDH and the time it takes to order and refill. This leads to delays or omissions in patients receiving oxygen.
2. Oxygen cylinders are often large, heavy and difficult for staff to move within the facility and potentially hazardous when not attached to wall braces, as is often the case.
3. Annually, the Fiji MoHMS spends approximately 2.5 million dollars on the supply of oxygen from one provider.

From the above three issues, critical success factors needed for the assessment of a preferred option of delivery of oxygen in Fiji must include availability, safety, ease of use, feasibility, and affordability.

The preferred option is Option Three which is a combination of a minimum number of concentrators with a cylinder as back-up (Table 1). The whole of life cost for this Option is $434,032 over 5 years, with $202,340 required for capital costs and $231,692 for operating costs, compared to $793,004 for the status quo. This Option is preferred due to affordability compared to the status quo and meeting the most critical success factors. Although Option Two had the highest net present value, we were informed by staff in the sites that having cylinder oxygen as a back-up is important. Option Four may be preferable to Option Three where, for example, the nature of the facility makes one or more extra concentrators preferable from a practical point of view. Regardless of the more expensive capital costs to set up a concentrator-based oxygen system, the operating costs over 5 years were less and therefore the cost analysis shows that all options that use concentrators are cheaper than using a system with only oxygen cylinders.

**Table 1:** Analysis of the short-list options for oxygen systems in Fiji’s 16 Sub-Divisional Hospitals

	Option One: Cylinders only (status quo)	Option Two: Concentrators only	Option Three: Minimum number of concentrators with cylinder backup	Option Four: Convenient number of concentrators with cylinder backup
Appraisal period	Five years	Five years	Five years	Five years
Capital costs	$75,912	$187,157	$202,340	$242,945
Operating costs	$717,091	$72,568	$231,692	$231,692
Whole of life costs	$793,004	$259,725	$434,032	$474,637
Whole of life savings	–	$558,151	$358,972	$318,366
Cost-benefit analysis of monetary costs and benefits				
Present value of monetary benefits	–	$573,861	$432,182	$432,182
Present value of costs	–	$111,245	$126,427	$167,033
Net present value	–	$462,616	$305,755	$265,149
**Multi-criteria analysis of non-monetary benefits: critical success factors**^**a**^				
Oxygen availability	**+**	**+**	**++**	**++**
Safety	**+**	**++**	**+**	**+**
Ease of use	**–**	**++**	**++**	**++**
Feasibility	**+**	**–**	**++**	**++**
Affordability	**–**	**++**	**++**	**++**

^a^Key

**-** Does not meet critical success factor

**+** Meets the critical success factor partly

**++** Meets the critical success factor completely

After undertaking sensitivity analyses, the whole of life cost for the preferred option ranged from $327,940 to $506,920 (Table 2). The estimated cost of the preferred option was 55% that of the status quo, with a range of 41 to 64% in the best and worst case scenarios, respectively.

**Table 2:** Sensitivity analyses results showing the best case, estimated case, and worst case costs and the proportion of costs compared to Option One (status quo)

	Best case		Estimated case		Worst case	
	Cost	^a^	Cost	^a^	Cost	^a^
Option One – cylinders only	–	–	$793,034	0.0	–	–
**Variables**						
Cylinders	$327,940	0.41	$434,032	0.55	$552,587	0.70
Concentrators	$412,580	0.52	$434,032	0.55	$454,398	0.57
Maintenance	$412,673	0.52	$434,032	0.55	$455,390	0.57
Transport	$398,358	0.50	$434,032	0.55	$469,705	0.59
Shipping	$412,163	0.52	$434,032	0.55	$506,920	0.64

^a^The cost of the best case, estimated case, and worst case is divided by the cost of the oxygen system that is currently in place (i.e. Option One - cylinders only)

## Discussion

Our results suggest that the installation of oxygen concentrators into sub-divisional hospitals in Fiji would be cost saving for the Fijian MoHMS. Moreover, it would help ensure that oxygen is continuously available, safer and easier to use, which is not currently the case. However, to realise these benefits, installation of concentrators would need to be optimised through professional installation, adequate staff training and good management and oversight by all related parties such as the Fiji MoHMS, clinical and biomedical engineering staff, and donor organisations.

Studies undertaken in other LMICs have reported clinical and financial benefits of using oxygen concentrators [3, 5, 8, 12, 13, 15, 22–25]. Any installation of concentrators, however, needs to be supported with technical training, maintenance and monitoring [16]. This was evident in a study in the Gambia where the authors reported a high percentage of concentrators functioning, beyond their standard 5 years, with routine preventive maintenance that was low cost and required a low level of technical experience [24]. In contrast, monitoring and evaluation data from Nigeria found only two of the 57 concentrators installed remained fit for use, with most producing insufficient concentrations of oxygen to meet medical standards [26]. This highlights the importance of good training not only of technical staff maintaining concentrators but also of health care staff administering the oxygen, and good systems for maintenance. Difficulties with concentrators were also reported in Laos with 37, 18 and 34% of concentrators requiring repair at 12, 24 and 40 months, respectively, and that significant local engineering capacity was required to address ongoing issues [27]. This need for local engineering capacity was also identifed in the Gambian study [28]. In recognition of the growing interest in oxygen concentrators and their use in LMICs, the WHO has prepared a technical report to guide users [16, 29].

While every effort was used to obtain reliable information for our analysis, the data have limitations. Data from the analysis of three pilot sites were used to calculate the costs of changes to the oxygen system in all SDHs therefore assumptions were required which may not be accurate for other sites. However, adjusting rates to show the best and worst case cost scenarios allowed for these variations and continued to show a cost benefit for the preferred option even with the worst case cost scenario. Oxygen use before the introduction of concentrators in the three sites is likely to have been rationed and to have increased after the introduction of concentrators. This is likely to make cylinder supplies appear less costly than if demand and supply parameters were applied identically. This adds additional reassurance that the findings in favour of concentrator-based supply solutions are robust. Our analysis was limited to SDHs and is therefore not indicative for the whole of the health services in Fiji. Neither is it indicative of costs in hospitals related to anaesthesia and intensive care, where high flows are used. Economies of scale and logistical considerations mean that supplying oxygen by concentrators in larger Divisional Hospitals or supplying facilities smaller than SDHs may vary to what is reported here. Oxygen demand within a country is difficult to estimate [30] particularly as it is often difficult to collect long term data, and there are also seasonal fluctuations as well as major health-impacting events such as hurricanes and epidemics. We have provided a best estimate on the data available, but oxygen usage as well as supply, procurement, and costs of both concentrators and cylinder oxygen may change over time. Lastly, we used an investment logic mapping exercise which may not have been as robust as other models, such as multi-criteria decision analysis [31, 32], which may have therefore underestimated the problems identified as critical success factors.

Installing and using oxygen concentrators in a country’s health system is not without its challenges. The warranty of the concentrators is for three of their expected 5-year lifespan, therefore the role of biomedical technicians is critical, as is having the relevant supply of spare parts, robust maintenance systems, and a back-up supply of cylinder oxygen. Changing staff behaviour to encourage them to use the concentrators is also a potential challenge, however, from discussion with staff in the sites we know that with good training staff prefered to use the concentrators. It is also worth considering where the funding for concentrators should come from, and in a study of the cost effectiveness of using oxygen concentrators in Papua New Guinea the authors reported them to be the “best-buy” for supply of oxygen that could be part of a national supply strategy rather than relying on donor support [11]. To achieve long-term sustainability, introduction of oxygen concentrators needs to be undertaken by the national Ministry of Health, in partnership with other key stakeholder groups, and users, to ensure ownership is at the local level.

An option not considered here is larger oxygen ‘plants’, which are based on the same principles as oxygen concentrators, used on a larger scale to supply piped oxygen systems in hospitals or to fill cylinders. The cost analyses for such plants appear favourable, but there are operational hurdles to their success in practice, particularly in LMICs [33]. These include technical complexity, lack of standardisation of equipment, problematic after-sales support, and, when used to fill cylinders for distribution, continued exposure to the logistical and safety concerns associated with oxygen cylinders. Such systems can form one component of a national system, where these hurdles have been adequately considered and addressed, and planning tools such as produced by UNICEF include this option [34].

Integrated national planning for oxygen supply and use, rather than ad hoc solutions at facility or health district level, is needed to optimise both availability for hypoxaemic patients and cost. Ensuring access to oxygen is likely to cost less per patient in large facilities than in small ones, but planning access nationally can prevent this becoming a diversion or barrier, by making the costs of universal access more transparent and able to be compared to the status quo.

The current COVID-19 global pandemic has put the spotlight on emergency respiratory care, a fundamental component of which is access to medical oxygen. This analysis shows that in the Fijian context concentrator-based solutions are likely to provide security of supply that meets the needs of end users and suppliers. This is very likely to also be the case in other similar LMIC contexts, which are being disproportionately impacted by the current pandemic.

## Supplementary Information

**Additional file 1.** Topic guide for discussion with clinical and administrative staff.

**Additional file 2.** Assumptions.

**Additional file 3.** Acceptability of oxygen systems by health staff in sub-divisional hospitals in Fiji: Investment logic map.

## Data Availability

The datasets used and/or analysed during the current study are available from the corresponding author on reasonable request.

## Abbreviations

FiO_2_: Fiji Islands Oxygen Project
FJD: Fijian dollar
HC: Health Centres
ILM: Investment Logic Mapping
LMIC: Low and middle-income countries
MoHMS: Ministry of Health and Medical Services
NPV: Net present value
NS: Nursing Stations
OC: Oxygen concentrators
PV: Present value
PVC: Present value of costs
SDH: Sub-Divisional Hospitals
UNICEF: United Nations Children’s Fund
US: United States
WHO: World Health Organization
