# Supplementary material for: Cost analysis and critical success factors of the use of oxygen concentrators versus cylinders in sub-divisional hospitals in Fiji
Source: BMC Health Serv Res. 2021 Jul 2;21:636. doi: 10.1186/s12913-021-06687-8 (PMC8249838; doi:10.1186/s12913-021-06687-8)
Supplement: Supplementary file 1 — Additional file 1. Topic guide for discussion with clinical and administrative staff. [file 12913_2021_6687_MOESM1_ESM.docx]

**Additional file 1: Topic guide for discussion with clinical and administrative staff**

- What is your experience of using oxygen sources, including oxygen cylinders and oxygen concentrators?
- What are the main benefits of each of these oxygen sources?
- Have you experienced difficulties using any of these oxygen sources?
- What training have you received in using these oxygen sources?
- How available is oxygen in your facility?
- Are there any safety issues you have experienced with these oxygen sources?
- What is your understanding of the costs related to these different sources?
- Do you have any other comments regarding the use of oxygen from different sources in your health facility?
