# Supplementary material for: Cost analysis and critical success factors of the use of oxygen concentrators versus cylinders in sub-divisional hospitals in Fiji
Source: BMC Health Serv Res. 2021 Jul 2;21:636. doi: 10.1186/s12913-021-06687-8 (PMC8249838; doi:10.1186/s12913-021-06687-8)
Supplement: Supplementary file 2 — Additional file 2. Assumptions. [file 12913_2021_6687_MOESM2_ESM.docx]

**Additional file 2: Assumptions**

| **Capital costs** |  |
| --- | --- |
| **Concentratators** | - The cost of 10L Intensity concentrators ($1,122 per item) was sourced from a manufacturer’s invoice. The cost was multiplied by the number of concentrators required by each site. - The minimum number of concentrators required to still be able to treat all patients that required oxygen, was calculated. In contrast, the pilot sites were installed with the number of concentrators that were convenient to the clinical staff. - For the remaining 13 SDHs, the minimum number was based on a WHO guideline of 1 concentrator per 15 beds and an additional spare concentrator per facility (*World Health Organization. The clinical use of oxygen in hospitals with limited resources. Guidelines for health-care workers, hospital engineers and programme managers, 2009 (page 91).* |
| **Spare parts** | - Spare parts details (listed below) for the concentrators were sourced from a recommended list of spare parts for 21 concentrators (*World Health Organization. The clinical use of oxygen in hospitals with limited resources. Guidelines for health-care workers, hospital engineers and programme managers, 2009 (page 41)*. These costs were for 5 years, however the concentrators have a 3 year warranty. Therefore, we calculated the cost for two years of spare parts required for each concentrator. - The cost of spare parts was multiplied by the number of concentrators at each SDH.  \| Item description \| Cost per item (USD) \| Number  required \| \| --- \| --- \| --- \| \| Filter, inlet \| $1.14 \| 20 \| \| Filter intake resonator \| $3.43 \| 10 \| \| 9V Battery bracket \| $2.1 \| 2 \| \| Screw, 9V battery bracket \| $0.22 \| 2 \| \| Diss O_2_ outlet \| $1.14 \| 10 \| \| Diss fitting, O_2_ outlet \| $2.85 \| 5 \| \| Tubing \| $0.44 \| 500m \| \| Check valve \| $5.25 \| 20 \| \| Valve, solenoid, 1/4", 2200VDC \| $25.2 \| 2 \| \| Flow meter \| $57.06 \| 10 \| \| Circuit board \| $68.47 \| 8 \| \| Circuit board O_2_ monitor \| $143.98 \| 8 \| \| Sieve beds \| $284.52 \| 21 \| \| Compressor assy \| $370.88 \| 10 \| \| Compressor kit \| $62.77 \| 12 \| \| Alarm buzzer with harness \| $22.83 \| 2 \| \| Capacitors \| $11.41 \| 5 \| \| 9V batteries \| $3.15 \| 12 \| |
| **Shipping** | - The international ($12,868) and internal ($9000) shipping was sourced from Cure Kids records of shipping costs for the pilot sites. The shipping cost was included as a total for the three sites. - The preferred option will aim to have the concentrators for all 13 SDHs shipped at one time. - Therefore, the cost the total cost of shipping to the pilot sites was divided by thirteen and applied to each of the 13 SDHs. |
| **Solar power system** | - Solar power system costs were sourced from an invoice from the manufacturers. Cure Kids NZ provided us with a list of the SDHs and the status of their electricity availability. - These costs were applied to three SDHs that do not have a reliable power source. |
| **Cylinder equipment** | - Cylinders require regulators, humidifiers, and flow meters for use in health care. The costs for the equipment were sourced from Fiji Pharmaceutical and Biomedical Services (FPBS). - For the preferred option, each site will only have one cylinder. Therefore, the costs for one set of the equipment was applied to each site. |
| **Ongoing costs** |  |
| **Electricity** | - Electricity costs were calculated using the wattage of the 10L concentrators, the cost of power per kWH ($0.4), and the number of hours per month of concentrator usage. The wattage of the concentrators was sourced from the manufacturer’s website, and the cost of power was sourced from an electricity company invoice from Nausori Health Centre. - The estimated concentrator hours per day from Taveuni site was 6.3 hours for this 33 bed facility. We categorised concentrator running time per day according to the number of beds per SDH. Taveuni was used as our reference category (rounded to 6 hours usage per day) then subtracted/added one hour per day according to bed category. - Electricity costs were not calculated for the sites that had a solar power system. |
| **Cylinder refill and rental** | - Refill and rental costs per month at the pilot sites were sourced from invoices and provided by the office clerks at each site. However, as the invoices included the costs for cylinders used at HCs and NS in the sub-divisions, we could not use these costs. - Refill and rental costs per cylinder were provided by the office clerks at Nabouwalu Hospital and Taveuni Hospital. - We assumed that each of the SDHs need one size G cylinder per month as back-up. The refill and rental cost of one size G cylinder per month for 5 years was included in calculations for each of the 16 SDHs. |
| **Transport for cylinder refill** | - Transport costs for cylinders at Nausori Health Centre and Nabouwalu Hospital were calculated using the fuel costs per month supplied by the office clerks, and the hourly rate of a driver supplied by Nausori Health centre office clerk. We assumed the hourly rate of a driver would be the same for all of Fiji. - Taveuni Hospital did not have transport costs as Oceania Gas provided refill for the Hospital. - As we do not know any information on the transport arrangements for the remaining SDHs, we calculated the average cost for the two pilot sites we have transport data for, and applied this cost to the other SDHs. |
| **Maintenance by biomedical engineers** | - Cure Kids provided the approximate salary of a biomedical engineer. - Maintenance is required for both cylinder and concentrator systems. The schedule for maintenance at each site by biomedical engineers has not yet been confirmed, however we have assumed that one day every 6 months with two extra call-outs per site will be appropriate. From the yearly salary, we calculated the cost for four days of work per year. |
