# Supplementary material for: Cost analysis and critical success factors of the use of oxygen concentrators versus cylinders in sub-divisional hospitals in Fiji
Source: BMC Health Serv Res. 2021 Jul 2;21:636. doi: 10.1186/s12913-021-06687-8 (PMC8249838; doi:10.1186/s12913-021-06687-8)

**Additional file 3**: Acceptability of oxygen systems by health staff in sub-divisional hospitals in Fiji: Investment logic map


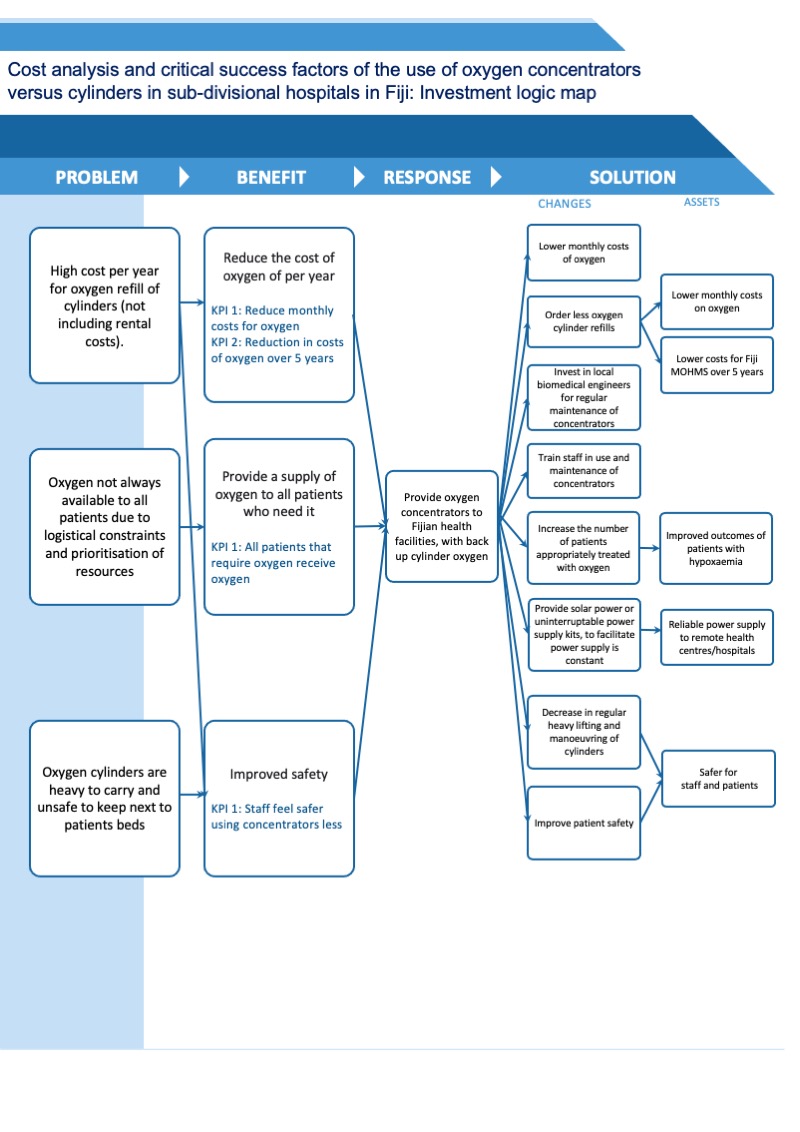

Supplement: Supplementary file 3 — Additional file 3. Acceptability of oxygen systems by health staff in sub-divisional hospitals in Fiji: Investment logic map. [file 12913_2021_6687_MOESM3_ESM.docx]
